# Association between minerals intake and childhood obesity: A cross-sectional study of the NHANES database in 2007–2014

**DOI:** 10.1371/journal.pone.0295765

**Published:** 2023-12-27

**Authors:** Lu Wang, Wei Liu, Sitong Bi, Li Zhou, Lihua Li

**Affiliations:** Department of pediatrics, Beijing Luhe Hospital Affiliated to Capital Medical University, Beijing, P.R. China; Florida State University, UNITED STATES

## Abstract

**Background:**

The roles of minerals in obesity received increasing attention recently due to its oxidant or antioxidant functions and effects on insulin and glucose metabolism that may be associated with obesity. Herein, this study aims to explore the association between minerals and obesity and body mass index (BMI) in children with different ages, and hope to provide some references for prevention and management in children with high-risk of obesity.

**Methods:**

Data of children aged 2–17 years old were extracted from the National Health and Nutrition Examination Survey (NHANES) database in 2007–2014 in this cross-sectional study. Weighted univariate and multivariate logistic regression and liner regression analyses were used to screen covariates, and explore the association between minerals [including calcium (Ca), phosphorus (P), magnesium (Mg), iron (Fe), zinc (Zn), copper (Cu), sodium (Na), potassium (K) and selenium (Se)] and childhood obesity and BMI. The evaluation indexes were β, odds ratios (ORs) and 95% confidence intervals (CIs). These relationships were also investigated in age subgroups.

**Results:**

Among 10,450 eligible children, 1,988 (19.02%) had obesity. After adjusting for covariates, we found the highest quartile of dietary Fe [OR = 0.74, 95%CI: (0.58, 0.95)] and Zn [OR = 0.70, 95%CI: (0.54, 0.92)] intakes were associated with low odds of childhood obesity, while that of dietary Na intake seemed to be positively linked to childhood obesity [OR = 1.35, 95%CI: (1.05, 1.74)]. High dietary intakes of Ca, Na and K were positively associated with children’s BMI, on the contrary, dietary Fe and Zn consumptions had a negative one (all *P*<0.05). Additionally, these associations were also found in children with different age (all *P*<0.05).

**Conclusion:**

Dietary Fe and Zn intakes played positive roles in reducing childhood obesity or BMI, while the intakes of Na should be controlled suitably.

## Introduction

The global prevalence of childhood obesity has grown sharply in recent decades [1], and generated an enormous individual and socioeconomic burden [2,3]. Approximately 70 million children will be obese or overweight in developing countries by 2025 [4]. Obesity in children has contributed to the increased risk of chronic diseases, such as obesity in adulthood, mental health problems, diabetes mellitus (DM), cardiovascular disease (CVD), some types of cancer, and death [5]. Therefore, the prevention and treatment strategies on childhood obesity need to be given primary importance.

Dietary/nutritional intervention plays a central role in the prevention of obesity [6]. In the past, most dietary measures on weight control focused on reducing the intake of macronutrients such as carbohydrates and fats [7,8]. Recently, the effects of minerals on obesity have received increasing attention [9]. Minerals can alter the composition of the intestinal microbiota, gut barrier function, compartmentalized metabolic inflammation, cellular glucose transport, and endocrine control of glucose metabolism, which may be associated with the occurrence and development of obesity [10,11]. Transition metals such as iron (Fe), zinc (Zn), copper (Cu) and selenium (Se) play important roles in cell metabolism, and their oxidant/antioxidant functions may be involved in the mechanism of obesity [12]. Also, sodium (Na) as a macroelement has been reported to be positively associated with the risk of obesity [13], which may affect insulin and glucose metabolism, accelerate leptin production or secretion and enhance leptin resistance, leading to energy imbalance, accumulation of adipose tissue mass and eventually obesity [14]. Gu et al. [15,16] found that serum Zn levels in children with obesity were significantly lower than those without obesity, while the serum Cu levels were higher. However, studies extensively discussing the association between dietary minerals intake and the risk of childhood obesity are still absent.

Herein, this study based on the National Health and Nutrition Examination Survey (NHANES) database, with the aim of exploring association between nine common minerals including calcium (Ca), phosphorus (P), magnesium (Mg), Fe, Zn, Cu, Na, potassium (K) and Se, and obesity and body mass index (BMI) in children, in order to provide some dietary references for prevention and management of childhood obesity.

## Methods

### Study design and population

Data of children in this cross-sectional study were extracted from the NHANES database in 2007–2014. The NHANES is a multipurpose research program done by the National Center for Health Statistics (NCHS) to assess the health and nutritional status of population in the United States [17]. It collects data of approximately 5,000 persons from 15 areas regularly since 1999 that includes a household interview followed by a standardized physical examination in a mobile examination center (MEC). A stratified multistage sampling design with a weighting scheme based on the selection of counties, blocks, households, and persons within households is used by NHANES to represent the civilian, non-institutionalized population in the United States and accurately estimate disease prevalence (https://www.cdc.gov/nchs/nhanes/index.htm).

We initially included 13,058 children aged 2–17 years old from the database. Then, those who without information of minerals intake, BMI, or poverty income ratio (PIR) were excluded. Finally, 10,450 children were eligible. The NHANES survey was approved by the institutional review board (IRB) of NCHS. The participants’ legal guardians/next of kin have provided written informed consent for participation. Since all the data were de-identified and publicly available, no ethical approval from the IRB of Beijing Luhe Hospital Affiliated to Capital Medical University was required.

### Assessment of dietary intake of minerals

NHANES collects the dietary status of participants through two 24-hour dietary recall interviews, which is according to the United States Department of Agriculture (USDA) automated multiple-pass method (AMPM) [18]. The first dietary recall interview was conducted in person, and the second one was conducted 3–10 days later via a phone call. During the interviews, consumption frequency, duration, and dosage were recorded for each of the dietary, dietary supplements, and prescription medication over the prior 30 days using questionnaires, which can be used for calculation of the average daily intake of nutrients. Respondents for the dietary interviews included the following: a proxy for child aged <6 years old; a proxy with the assistance of the child for those aged from 6 to 8 years old; assistance of a proxy for child aged 9–11 years old; and children aged ≥12 years old who answered by themselves.

In the current study, we extracted the data on dietary mineral intake and its supplements in the first 24-hour recall, and standardized by the total energy intake: mineral intake/total energy (mg/1000kcal). The dietary Ca (mg), P (mg), Mg (mg), Fe (mg), Zn (mg), Cu (mg), Na (mg), K (mg) and Se (mcg) intakes were divided into four levels according to their respective quartiles.

### Obesity diagnosis and BMI measurement

The children BMI was calculated using the formula: weight/height^2^ (kg/m^2^). Obesity was judged by the BMI z-score, the CDC recommended percentiles, which was calculated accounting for age and sex. A BMI z-score ≥95th percentile indicates obesity. For more details of the calculation of the BMI z-score please visit the NHANES website: https://www.cdc.gov/healthyweight/assessing/bmi/childrens_bmi/about_childrens_bmi.html.

### Covariates

We also collected variables including age, gender, race, PIR, physical activity, sedentary time, maternal smoking during pregnancy, cotinine (ng/mL), and the intakes of protein (gm/1000kcal), carbohydrate (gm/1000kcal) and fat (gm/1000kcal) from the database. Physical activity of children aged 12–17 years old was translated into energy expenditure. The Metabolic equivalent (MET) was calculated based on information collected from the NHANES questionnaire of physical activity reports (PAQ) [19]: Energy expenditure (MET·min) = recommended MET × exercise time of corresponding activity (min). The ideal physical activity was ≥180 MET·min/day (for children aged 12–17 years old or ≥60 min/day (for children aged 2–11 years old), and otherwise recognized as not achieve the ideal physical activity. Sedentary time (time watching TV or video or using a computer) per average day over the last 30 days was collected through the household interview. The sedentary time was divided into four levels: <3 hours, 3–6 hours, ≥6 hours and unknown. The detection limit of cotinine is 0.011 ng/mL. The intakes of total energy, carbohydrate, protein and fat were collected according to both food and supplements respectively from the first 24-hour dietary recall in the NHANES.

### Statistical analysis

The continuous variables were described using mean ± standard error (mean ± SE), and t test was used for comparation between groups. Categorical data were expressed using frequency and constituent ratio [N (%)], and chi-square test (χ^2^) was used for the comparison. A set of NHANES special weights “WTDRD1” were used for the analyses because we included data on the first 24-hour dietary recall in dietary minerals intake assessment. These weights were constructed by taking the 2-year cycle MEC sample weights (WTMEC2YR) and further adjusting for (a) the additional non-response and (b) the differential allocation by day of the week for the dietary intake data collection.

When explored the association between dietary minerals intake and childhood obesity, weighted univariate and multivariate logistic regression analyses were used. Adjusted model adjusted covariates including age, gender, race, PIR, physical activity, sedentary time, maternal smoking during pregnancy, cotinine, carbohydrate intake, protein intake and fat intake. The evaluation index was odds ratios (ORs) and 95% confidence intervals (CIs). When explored the association between dietary minerals intake and BMI weighted univariate and multivariate liner regression analyses were performed. Adjusted model adjusted covariates including age, gender, race, PIR, physical activity, sedentary time, and maternal smoking during pregnancy. The evaluation index was the estimated value (β) and 95% confidence intervals (CIs). We also explored these relationships in subgroups of different ages (1–5, 6–11, and 12–17 years old). The reference group in the dependent variable was the lowest quartile of each dietary mineral intake level.

Two-sided *P*<0.05 was considered significant. Statistical analysis was performed using SAS 9.4 (SAS Institute, Cary, NC, USA). Missing variables, including physical activity, sedentary time, maternal smoking during pregnancy, and cotinine, were classified into the “unknown” category.

## Results

### Characteristics of children

**Fig 1:**
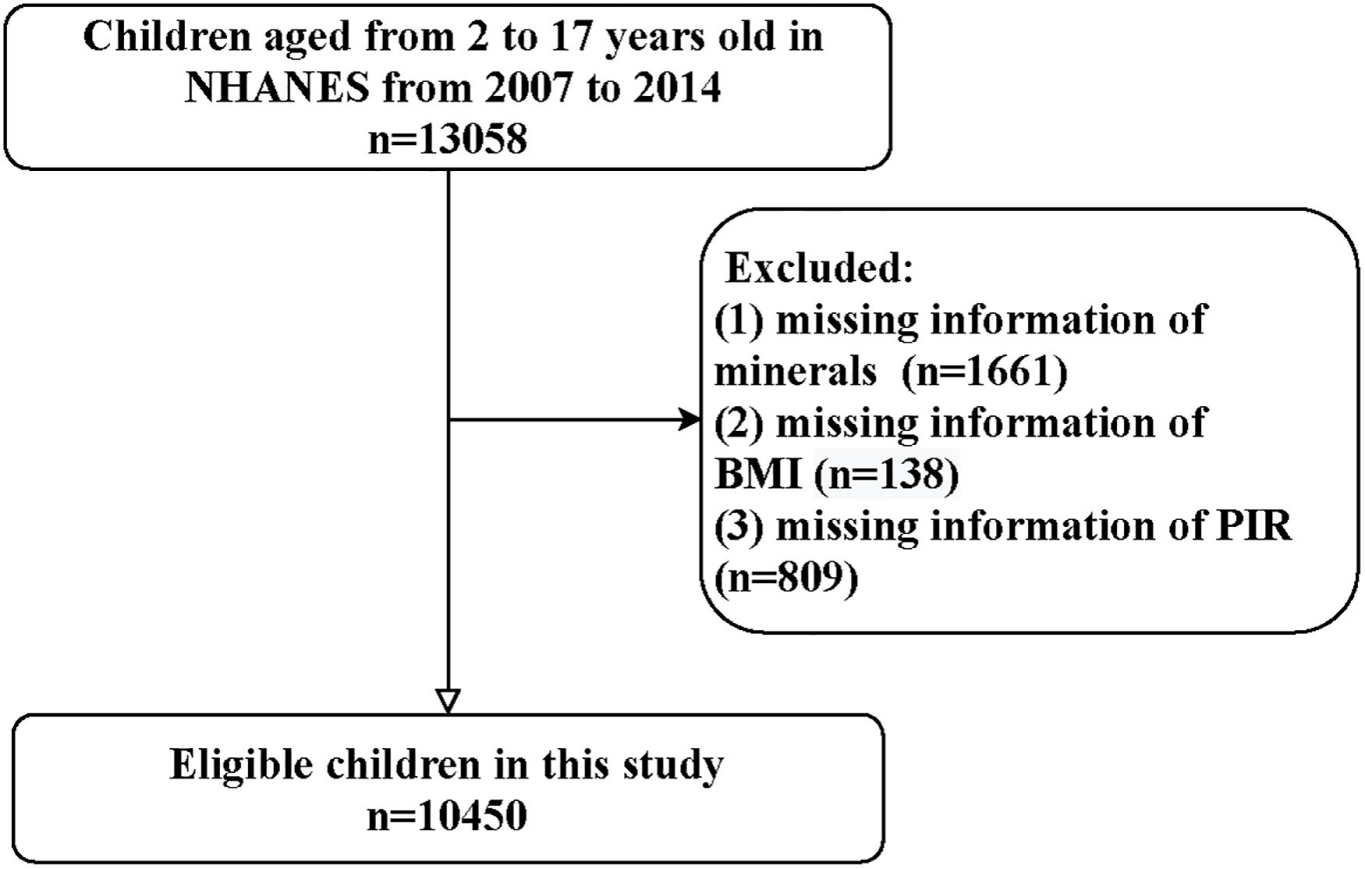
Flowchart of the participants screening.

Fig 1 is the flowchart of participants screening. There were 13,058 children aged 2–17 years old in the NHANES database in 2007–2014. Then, children without the information of minerals intake (n = 1661), BMI (n = 138), or PIR (n = 809) were excluded. Finally, 10,450 were eligible.

The characteristics of eligible children are showed in Table 1. Among the eligible children, 1,988 (19.02%) had obesity. The average age of total children was 9.56 years old. More than half of children with obesity were male [1048 (53.49%)], while those without obesity included a higher percentage of females [4150 (50.12%)]. In children without obesity, 4,126 (44.74%) had an ideal physical activity level, while this number in children with obesity was 741 (31.90%). The dietary intake levels of Ca (578.39 vs. 559.39 mg/1000kcal), Fe (8.30 vs. 7.65 mg/1000kcal), Zn (6.15 vs. 5.79 mg/1000kcal), Cu (0.62 vs. 0.58 mg/1000kcal), Na (1572.30 vs. 1645.84 mg/1000kcal), and Se (49.35 vs. 50.92 mcg/1000kcal) were significantly different between non-obesity group and obesity group. In addition, race, PIR, sedentary time, maternal smoking during pregnancy, cotinine, and intakes of protein and carbohydrate were also significantly different between non-obesity group and obesity group (all *P*<0.05).

**Table 1:** Characteristics of eligible children.

Variables	Total (n = 10450)	Non-obesity (n = 8462)	Obesity (n = 1988)	Statistics	*P*
Age, years, Mean (S.E)	9.56 (0.08)	9.33 (0.08)	10.59 (0.14)	t = -8.86	<0.001
Age levels, n (%)				χ^2^ = 85.13	<0.001
2–5	2888 (24.25)	2564 (26.72)	324 (12.89)		
6–11	4172 (37.68)	3272 (36.58)	900 (42.73)		
12–17	3390 (38.07)	2626 (36.70)	764 (44.38)		
Gender, n (%)				χ^2^ = 3.99	0.046
Male	5360 (50.52)	4312 (49.88)	1048 (53.49)		
Female	5090 (49.48)	4150 (50.12)	940 (46.51)		
Race, n (%)				χ^2^ = 74.87	<0.001
Mexican American	2427 (14.25)	1880 (12.98)	547 (20.11)		
Other Hispanic	1171 (7.44)	908 (7.03)	263 (9.33)		
Non-Hispanic White	3099 (56.57)	2612 (58.38)	487 (48.23)		
Non-Hispanic Black	2600 (13.96)	2069 (13.58)	531 (15.72)		
Other Race—including multi-racial	1153 (7.77)	993 (8.03)	160 (6.62)		
PIR, Mean (S.E)	2.44 (0.07)	2.51 (0.07)	2.13 (0.08)	t = 4.57	<0.001
Physical activity, n (%)				χ^2^ = 49.47	<0.001
Ideal physical activity	4867 (42.45)	4126 (44.74)	741 (31.90)		
Not ideal physical activity	2570 (24.14)	1994 (22.97)	576 (29.52)		
Unknown	3013 (33.40)	2342 (32.28)	671 (38.58)		
Sedentary time levels, n (%)				χ^2^ = 61.84	<0.001
<3	1915 (17.08)	1654 (18.56)	261 (10.23)		
3–6	1227 (10.27)	984 (10.23)	243 (10.44)		
≥6	1955 (20.96)	1485 (19.47)	470 (27.80)		
Unknown	5353 (51.70)	4339 (51.73)	1014 (51.53)		
Maternal smoking during pregnancy, n (%)				χ^2^ = 10.42	0.005
No	8057 (73.94)	6574 (74.98)	1483 (69.13)		
Yes	1160 (11.99)	909 (11.37)	251 (14.89)		
Unknown	1233 (14.07)	979 (13.65)	254 (15.98)		
Cotinine, ng/mL, Mean (S.E)	4.53 (0.65)	4.55 (0.66)	4.44 (1.50)	t = 0.08	0.938
Cotinine levels, n (%)				χ^2^ = 40.02	<0.001
0.011	2047 (22.37)	1667 (22.84)	380 (20.21)		
>0.011	5696 (54.24)	4423 (52.04)	1273 (64.39)		
Unknown	2707 (23.38)	2372 (25.11)	335 (15.40)		
Ca, mg/1000kcal, Mean (S.E)	575.01 (5.13)	578.39 (5.31)	559.39 (7.98)	t = 2.47	0.016
P, mg/1000kcal, Mean (S.E)	677.59 (3.46)	678.24 (3.70)	674.61 (5.98)	t = 0.58	0.564
Mg, mg/1000kcal, Mean (S.E)	127.24 (0.73)	127.14 (0.76)	127.66 (1.67)	t = -0.30	0.767
Fe, mg/1000kcal, Mean (S.E)	8.18 (0.09)	8.30 (0.10)	7.65 (0.13)	t = 4.49	<0.001
Zn, mg/1000kcal, Mean (S.E)	6.09 (0.08)	6.15 (0.09)	5.79 (0.12)	t = 2.50	0.015
Cu, mg/1000kcal, Mean (S.E)	0.61 (0.01)	0.62 (0.01)	0.58 (0.01)	t = 2.57	0.013
Na, mg/1000kcal, Mean (S.E)	1585.40 (8.37)	1572.30 (9.77)	1645.84 (17.90)	t = -3.45	<0.001
K, mg/1000kcal, Mean (S.E)	1188.07 (6.81)	1185.58 (7.20)	1199.54 (15.65)	t = -0.85	0.398
Se, mcg/1000kcal, Mean (S.E)	49.63 (0.29)	49.35 (0.34)	50.92 (0.48)	t = -2.64	0.010
Protein, gm/1000kcal, Mean (S.E)	35.90 (0.19)	35.67 (0.22)	36.96 (0.29)	t = -3.77	<0.001
Carbohydrate, gm/1000kcal, Mean (S.E)	135.73 (0.44)	136.13 (0.48)	133.85 (0.87)	t = 2.37	0.021
Fat, gm/1000kcal Mean (S.E)	36.16 (0.15)	36.10 (0.17)	36.46 (0.31)	t = -1.07	0.291

t: Test, χ^2^: ^C^hi-square test.

S.E: Standard error, PIR: Poverty-income ratio, Ca: Calcium, P: Phosphorus, Mg: Magnesium, Fe: Iron, Zn: Zinc, Cu: Copper, Na: Sodium, K: Potassium and Se: Selenium.

### Association between dietary minerals intake and childhood obesity

We explored the association between dietary minerals intake and childhood obesity (Table 2). After adjusting for covariates, we found that compared with the lowest quartiles, the highest quartile of dietary Fe [OR = 0.74, 95% CI: (0.58, 0.95)] and Zn [OR = 0.70, 95% CI: (0.54, 0.92)] intakes were associated with lower odds of childhood obesity, while the highest quartile of dietary Na intake [OR = 1.35, 95% CI: (1.05, 1.74)] was associated with higher odds of childhood obesity.

**Table 2:** Association between dietary minerals intake and childhood obesity.

Dietary minerals intake	Children with obesity/total samples	Crude model OR (95% CI)	Adjusted model^#^ OR (95% CI)
Ca (mg/1000kcal)			
≤391.56	580 / 2939	Ref	Ref
391.56–544.69	521 / 2687	0.94 (0.76–1.16)	0.98 (0.78–1.23)
544.69–721.81	485 / 2473	0.95 (0.78–1.17)	1.04 (0.85–1.28)
>721.81	402 / 2351	0.82 (0.68–1.00)	0.99 (0.79–1.25)
P (mg/1000kcal)			
≤549.25	578 / 2829	Ref	Ref
549.25–661.63	502 / 2777	0.81 (0.68–0.96) *	0.76 (0.63–0.92) **
661.63–786.65	500 / 2545	1.03 (0.85–1.25)	1.02 (0.82–1.28)
>786.65	408 / 2299	0.92 (0.78–1.09)	0.92 (0.70–1.21)
Mg (mg/1000kcal)			
≤100.41	555 / 2770	Ref	Ref
100.41–122.18	494 / 2673	0.98 (0.78–1.23)	1.04 (0.81–1.33)
122.18–146.32	475 / 2547	1.03 (0.84–1.28)	1.13 (0.90–1.42)
>146.32	464 / 2460	0.97 (0.77–1.22)	1.09 (0.85–1.39)
Fe (mg/1000kcal)			
≤5.39	554 / 2618	Ref	Ref
5.39–6.85	495 / 2562	0.98 (0.81–1.18)	0.97 (0.80–1.19)
6.85–9.33	508 / 2637	0.91 (0.75–1.11)	0.88 (0.72–1.09)
>9.33	431 / 2633	0.73 (0.57–0.92) **	0.74 (0.58–0.95) *
Zn (mg/1000kcal)			
≤4.01	560 / 2771	Ref	Ref
4.01–5.20	514 / 2646	0.88 (0.74–1.03)	0.85 (0.72–1.01)
5.20–7.08	490 / 2551	0.91 (0.76–1.10)	0.86 (0.67–1.11)
>7.08	424 / 2482	0.75 (0.61–0.94) *	0.70 (0.54–0.92) *
Cu (mg/1000kcal)			
≤0.41	524 / 2805	Ref	Ref
0.41–0.50	523 / 2558	1.23 (0.98–1.54)	1.28 (1.02–1.61) *
0.50–0.64	548 / 2760	1.20 (0.93–1.54)	1.29 (1.01–1.66) *
>0.64	393 / 2327	0.89 (0.69–1.15)	1.04 (0.80–1.37)
Na (mg/1000kcal)			
≤1282.86	460 / 2664	Ref	Ref
1282.86–1541.84	489 / 2703	1.22 (0.95–1.57)	1.16 (0.90–1.50)
1541.84–1819.50	462 / 2564	1.13 (0.91–1.40)	1.01 (0.80–1.27)
>1819.50	577 / 2519	1.60 (1.28–2.01) ***	1.35 (1.05–1.74) *
K (mg/1000kcal)			
≤916.70	526 / 2693	Ref	Ref
916.70–1141.73	533 / 2662	1.15 (0.93–1.42)	1.17 (0.94–1.46)
1141.73–1407.27	460 / 2554	1.07 (0.86–1.34)	1.20 (0.95–1.53)
>1407.27	469 / 2541	1.10 (0.89–1.35)	1.27 (1.00–1.61)
Se (mcg/1000kcal)			
≤37.75	463 / 2580	Ref	Ref
37.75–48.05	484 / 2773	1.08 (0.84–1.38)	1.01 (0.77–1.33)
48.05–58.52	508 / 2501	1.23 (0.96–1.58)	1.10 (0.82–1.47)
>58.52	533 / 2596	1.30 (1.04–1.62) *	1.05 (0.78–1.40)

OR: Odd ratio, CI: Confidence interval, Ca: Calcium, Ref: Reference, P: Phosphorus, Mg: Magnesium, Fe: Iron, Zn: Zinc, Cu: Copper, Na: Sodium, K: Potassium and Se: Selenium

*P<0.05,

**P<0.01,

***P<0.001.

^#^ Adjusted for age, gender, race, PIR, physical activity, sedentary time, maternal smoking during pregnancy, cotinine, carbohydrate intake, protein intake and fat intake.

Note: We used g/1000kcal as the units of Ca, P, Mg, Na and K to expanded the value.

### Association between dietary minerals intake and childhood obesity in age subgroups

Table 3 shows the association between dietary minerals intake and childhood obesity in age subgroups. The highest quartile of dietary intake of Zn was associated with lower odds of childhood obesity in children aged 6–11 years old [OR = 0.54, 95% CI: (0.36, 0.80)], compared with the lowest quartile. Differently, higher level of Mg [OR = 1.56, 95% CI: (1.05, 2.32)] and Na [OR = 1.58, 95% CI: (1.11, 2.26)] consumptions were associated with higher odds of childhood obesity. In children aged 2–5 years old or 12–17 years old, these relationships were not significantly.

**Table 3:** Association between dietary minerals intake and childhood obesity in age subgroups.

Dietary minerals intake	2–5 years old OR (95% CI)	6–11 years old OR (95% CI)	12–17 years old OR (95% CI)
Ca (mg/1000kcal)			
≤391.56	Ref	Ref	Ref
391.56–544.69	0.94 (0.56–1.58)	0.98 (0.72–1.33)	0.95 (0.67–1.34)
544.69–721.81	0.78 (0.44–1.36)	1.03 (0.78–1.36)	1.09 (0.79–1.51)
>721.81	0.80 (0.45–1.42)	0.95 (0.63–1.43)	1.11 (0.74–1.65)
P (mg/1000kcal)			
≤549.25	Ref	Ref	Ref
549.25–661.63	0.72 (0.39–1.33)	0.89 (0.68–1.16)	0.64 (0.44–0.92) *
661.63–786.65	0.72 (0.41–1.25)	0.99 (0.73–1.33)	1.14 (0.81–1.63)
>786.65	0.68 (0.35–1.32)	0.88 (0.60–1.30)	1.01 (0.65–1.57)
Mg (mg/1000kcal)			
≤100.41	Ref	Ref	Ref
100.41–122.18	0.93 (0.58–1.51)	1.11 (0.83–1.49)	1.08 (0.70–1.67)
122.18–146.32	0.87 (0.52–1.47)	1.11 (0.76–1.60)	1.43 (1.00–2.04)
>146.32	0.93 (0.54–1.60)	1.56 (1.05–2.32) *	0.98 (0.66–1.45)
Fe (mg/1000kcal)			
≤5.39	Ref	Ref	Ref
5.39–6.85	0.72 (0.45–1.14)	1.35 (0.98–1.87)	0.78 (0.55–1.11)
6.85–9.33	0.61 (0.40–0.93) *	1.18 (0.86–1.62)	0.75 (0.55–1.01)
>9.33	0.62 (0.38–1.00)	0.97 (0.66–1.43)	0.66 (0.43–1.00)
Zn (mg/1000kcal)			
≤4.01	Ref	Ref	Ref
4.01–5.20	1.03 (0.68–1.55)	0.69 (0.47–1.00)	0.97 (0.67–1.42)
5.20–7.08	1.14 (0.75–1.74)	0.80 (0.57–1.13)	0.91 (0.56–1.46)
>7.08	0.80 (0.49–1.30)	0.54 (0.36–0.80) **	0.93 (0.61–1.42)
Cu (mg/1000kcal)			
≤0.41	Ref	Ref	Ref
0.41–0.50	1.31 (0.84–2.04)	1.49 (1.10–2.00) *	1.05 (0.72–1.53)
0.50–0.64	1.30 (0.87–1.94)	1.62 (1.12–2.35) *	1.06 (0.73–1.54)
>0.64	1.03 (0.60–1.76)	1.29 (0.93–1.81)	0.89 (0.57–1.38)
Na (mg/1000kcal)			
≤1282.86	Ref	Ref	Ref
1282.86–1541.84	1.26 (0.82–1.94)	1.26 (0.89–1.77)	1.08 (0.71–1.64)
1541.84–1819.50	0.98 (0.58–1.66)	1.04 (0.77–1.41)	0.96 (0.67–1.36)
>1819.50	1.43 (0.87–2.33)	1.58 (1.11–2.26) *	1.20 (0.81–1.78)
K (mg/1000kcal)			
≤916.70	Ref	Ref	Ref
916.70–1141.73	1.23 (0.74–2.05)	1.17 (0.87–1.57)	1.16 (0.80–1.70)
1141.73–1407.27	0.84 (0.49–1.46)	1.16 (0.81–1.65)	1.46 (1.03–2.09) *
>1407.27	1.20 (0.75–1.92)	1.44 (0.98–2.11)	1.19 (0.81–1.76)
Se (mcg/1000kcal)			
≤37.75	Ref	Ref	Ref
37.75–48.05	1.02 (0.61–1.68)	0.98 (0.69–1.39)	1.04 (0.66–1.65)
48.05–58.52	0.94 (0.53–1.67)	1.03 (0.72–1.48)	1.18 (0.74–1.87)
>58.52	0.81 (0.47–1.40)	1.02 (0.65–1.61)	1.12 (0.71–1.75)

OR: Odd ratio, CI: Confidence interval, Ca: Calcium, Ref: Reference, P: Phosphorus, Mg: Magnesium, Fe: Iron, Zn: Zinc, Cu: Copper, Na: Sodium, K: Potassium and Se: Selenium.

*P<0.05,

**P<0.01,

***P<0.001.

Adjusted for gender, race, PIR, physical activity, sedentary time, maternal smoking during pregnancy, cotinine, carbohydrate intake, protein intake and fat intake.

Note: We used g/1000kcal as the units of Ca, P, Mg, Na and K to expanded the value.

### Association between dietary minerals intake and BMI

We also explored the association between dietary minerals intake and children’s BMI (Table 4). After adjusting for covariates, dietary intake of Ca [β = 0.50, 95% CI: (0.07, 0.94)], Na [β = 0.48, 95% CI: (0.04, 0.91)] and K [β = 0.62, 95% CI: (0.19, 1.05)] were all positively associated with BMI, while Fe [β = -0.78, 95% CI: (-1.17, -0.39)] and Zn [β = -0.56, 95% CI: (-1.01, -0.11)] had negative associations with BMI.

**Table 4:** Association between dietary minerals intake and BMI in children.

Dietary minerals intake	Crude model β (95% CI)	Adjusted model^#^ β (95% CI)
Ca (mg/1000kcal)		
≤391.56	Ref	Ref
391.56 to 544.69	-0.49 (-0.91, -0.07) *	0.10 (-0.25, 0.44)
544.69 to 721.81	-0.69 (-1.13, -0.25) **	0.24 (-0.15, 0.64)
>721.81	-1.13 (-1.61, -0.65) ***	0.50 (0.07, 0.94) *
P (mg/1000kcal)		
≤549.25	Ref	Ref
549.25 to 661.63	-0.54 (-0.87, -0.20) **	-0.36 (-0.72, 0.01)
661.63 to 786.65	-0.57 (-1.03, -0.10) *	0.10 (-0.34, 0.53)
>786.65	-0.59 (-1.01, -0.17) **	0.23 (-0.23, 0.68)
Mg (mg/1000kcal)		
≤100.41	Ref	Ref
100.41 to 122.18	-0.65 (-1.12, -0.17) **	-0.06 (-0.51, 0.40)
122.18 to 146.32	-0.71 (-1.22, -0.20) **	0.28 (-0.13, 0.69)
>146.32	-0.41 (-0.90, 0.08)	0.30 (-0.15, 0.76)
Fe (mg/1000kcal)		
≤5.39	Ref	Ref
5.39 to 6.85	-0.28 (-0.70, 0.15)	-0.30 (-0.67, 0.08)
6.85 to 9.33	-0.78 (-1.18, -0.38) ***	-0.50 (-0.82, -0.19) **
>9.33	-1.28 (-1.72, -0.84) ***	-0.78 (-1.17, -0.39) ***
Zn (mg/1000kcal)		
≤4.01	Ref	Ref
4.01 to 5.20	-0.59 (-1.05, -0.13) *	-0.16 (-0.50, 0.18)
5.20 to 7.08	-0.67 (-1.02, -0.32) ***	-0.21 (-0.64, 0.22)
>7.08	-0.99 (-1.46, -0.53) ***	-0.56 (-1.01, -0.11) *
Cu (mg/1000kcal)		
≤0.41	Ref	Ref
0.41 to 0.50	-0.02 (-0.57, 0.52)	0.22 (-0.16, 0.60)
0.50 to 0.64	-0.11 (-0.65, 0.43)	0.19 (-0.20, 0.58)
>0.64	-0.52 (-1.04, 0.01)	-0.08 (-0.46, 0.31)
Na (mg/1000kcal)		
≤1282.86	Ref	Ref
1282.86 to 1541.84	0.47 (-0.03, 0.96)	0.14 (-0.26, 0.53)
1541.84 to 1819.50	0.59 (0.21, 0.97) **	-0.15 (-0.46, 0.16)
>1819.50	2.01 (1.52, 2.51) ***	0.48 (0.04, 0.91) *
K (mg/1000kcal)		
≤916.70	Ref	Ref
916.70 to 1141.73	-0.43 (-0.79, -0.06) *	0.14 (-0.22, 0.50)
1141.73 to 1407.27	-0.69 (-1.13, -0.25) **	0.40 (0.02, 0.77) *
>1407.27	-0.89 (-1.33, -0.45) ***	0.62 (0.19, 1.05) **
Se (mcg/1000kcal)		
≤37.75	Ref	Ref
37.75 to 48.05	-0.11 (-0.58, 0.35)	-0.21 (-0.65, 0.23)
48.05 to 58.52	0.18 (-0.33, 0.68)	-0.34 (-0.85, 0.17)
>58.52	1.22 (0.70, 1.73) ***	-0.09 (-0.60, 0.42)

BMI: Body mass index, CI: Confidence interval, Ca: Calcium, Ref: Reference, P: Phosphorus, Mg: Magnesium, Fe: Iron, Zn: Zinc, Cu: Copper, Na: Sodium, K: Potassium and Se: Selenium.

*P<0.05,

**P<0.01,

***P<0.001.

^#^ Adjusted for age, gender, race, PIR, physical activity, sedentary time, and maternal smoking during pregnancy.

Note: We used g/1000kcal as the units of Ca, P, Mg, Na and K to expanded the value.

### Association between dietary minerals intake and BMI in age subgroups

These associations were further explored in age subgroups, and the results were showed in Table 5. In children aged 2–5 years old, we only found a negative association between dietary Fe intake and BMI [β = -0.29, 95% CI: (-0.56, -0.03)]. In those who aged 6–11 years old, dietary intake of Zn was negatively linked to BMI [β = -1.04, 95% CI: (-1.65, -0.43)], while dietary intake of Na was positively linked to BMI [β = 0.65, 95% CI: (0.05, 1.25)]. In the 12–17 years old group, dietary Fe intake was negatively associated with BMI [β = -1.31, 95% CI: (-2.11, -0.52)], and similarly to those who aged 6–11 years old, relationship between dietary Na intake and BMI was positive [β = 0.84, 95% CI: (0.01, 1.66)].

**Table 5:** Association between dietary minerals intake and BMI in age subgroups.

Dietary minerals intake	2–5 years old β (95% CI)	6–11 years old β (95% CI)	12–17 years old β (95% CI)
Ca (mg/1000kcal)			
≤391.56	Ref	Ref	Ref
391.56 to 544.69	-0.05(-0.32, 0.22)	-0.06(-0.45, 0.32)	0.12(-0.62, 0.85)
544.69 to 721.81	-0.06(-0.31, 0.19)	-0.04(-0.48, 0.40)	0.46(-0.36, 1.28)
>721.81	0.07(-0.18, 0.32)	-0.19(-0.85, 0.47)	0.78(-0.13, 1.69)
P (mg/1000kcal)			
≤549.25	Ref	Ref	Ref
549.25 to 661.63	-0.08(-0.37, 0.22)	-0.35(-0.77, 0.06)	-0.61(-1.36, 0.15)
661.63 to 786.65	-0.10(-0.36, 0.15)	-0.20(-0.77, 0.34)	0.18(-0.69, 1.05)
>786.65	0.02(-0.28, 0.32)	-0.62(-1.30, 0.07)	0.44(-0.50, 1.37)
Mg (mg/1000kcal)			
≤100.41	Ref	Ref	Ref
100.41 to 122.18	-0.06(-0.34, 0.23)	-0.07(-0.50, 0.37)	-0.18(-1.15, 0.80)
122.18 to 146.32	-0.12(-0.45, 0.21)	0.08(-0.49, 0.65)	0.59(-0.29, 1.47)
>146.32	0.02(-0.34, 0.38)	0.31(-0.27, 0.89)	0.10(-0.71, 0.92)
Fe (mg/1000kcal)			
≤5.39	Ref	Ref	Ref
5.39 to 6.85	-0.12(-0.37, 0.15)	0.14(-0.37, 0.64)	-0.63(-1.44, 0.18)
6.85 to 9.33	-0.27(-0.49, -0.06) *	-0.02(-0.39, 0.36)	-1.09(-1.70, -0.48) ***
>9.33	-0.29(-0.56, -0.03) *	-0.51(-1.04, 0.02)	-1.31(-2.11, -0.52) **
Zn (mg/1000kcal)			
≤4.01	Ref	Ref	Ref
4.01 to 5.20	0.23(-0.03, 0.48)	-0.39(-0.93, 0.15)	-0.22(-1.07, 0.63)
5.20 to 7.08	0.12(-0.14, 0.38)	-0.32(-0.91, 0.26)	-0.42(-1.34, 0.51)
>7.08	-0.06(-0.28, 0.16)	-1.04(-1.65, -0.43) **	-0.44(-1.37, 0.48)
Cu (mg/1000kcal)			
≤0.41	Ref	Ref	Ref
0.41 to 0.50	0.06(-0.16, 0.28)	0.49(0.04, 0.94) *	0.00(-0.87, 0.88)
0.50 to 0.64	0.00(-0.20, 0.20)	0.61(0.09, 1.13) *	-0.12(-0.92, 0.67)
>0.64	-0.10(-0.39, 0.19)	0.07(-0.41, 0.56)	-0.25(-1.06, 0.56)
Na (mg/1000kcal)			
≤1282.86	Ref	Ref	Ref
1282.86 to 1541.84	0.01(-0.23, 0.24)	0.31(-0.12, 0.75)	0.26(-0.72, 1.24)
1541.84 to 1819.50	0.04(-0.21, 0.30)	-0.24(-0.75, 0.27)	0.04(-0.65, 0.74)
>1819.50	0.12(-0.16, 0.41)	0.65(0.05, 1.25) *	0.84(0.01, 1.66) *
K (mg/1000kcal)			
≤916.70	Ref	Ref	Ref
916.70 to 1141.73	0.06(-0.25, 0.38)	0.03(-0.39, 0.46)	0.13(-0.64, 0.89)
1141.73 to 1407.27	-0.11(-0.36, 0.14)	0.04(-0.49, 0.57)	0.74(-0.03, 1.51)
>1407.27	0.07(-0.18, 0.32)	0.15(-0.49, 0.79)	0.54(-0.37, 1.45)
Se (mcg/1000kcal)			
≤37.75	Ref	Ref	Ref
37.75 to 48.05	-0.07(-0.31, 0.18)	-0.11(-0.58, 0.36)	-0.36(-1.37, 0.64)
48.05 to 58.52	-0.11(-0.49, 0.26)	-0.29(-0.79, 0.21)	-0.34(-1.49, 0.82)
>58.52	-0.08(-0.40, 0.25)	0.12(-0.52, 0.76)	-0.01(-1.05, 1.03)

BMI: Body mass index, CI: Confidence interval, Ca: Calcium, Ref: Reference, P: Phosphorus, Mg: Magnesium, Fe: Iron, Zn: Zinc, Cu: Copper, Na: Sodium, K: Potassium and Se: Selenium.

*P<0.05,

**P<0.01,

***P<0.001.

Adjusted for gender, race, PIR, physical activity, sedentary time, and maternal smoking during pregnancy.

Note: We used g/1000kcal as the units of Ca, P, Mg, Na and K to expanded the value.

## Discussion

This study explored the association between dietary intakes of nine common minerals and obesity and BMI in children. The results showed that higher level of dietary Fe and Zn intakes were associated with lower odds of childhood obesity. Oppositely, higher levels of dietary Cu and Na intakes seemed to associated with higher odds of obesity. Dietary intakes of Ca, Na and K were positively linked to the children’s BMI, whereas dietary Fe and Zn consumptions shared negatively associations with BMI. Additionally, these relationships were also found in children with different age.

Findings in the current study supported hypotheses that dietary intake of Fe and Zn levels may be potential protective factors in the development of childhood obesity, whereas Cu, Na, Ca, Mg and K played opposite roles. Fan et al. [20] explored the relationship between serum metallic elements and obesity in children in the United States, and found the highest quartile of Cu concentrations in blood with an obesity status, while a negative association existed between blood Zn and obesity. We came to the same conclusion with Fan et al. in this study, and however, we used the dietary intake of metallic elements other than the serum concentrations, and were standardized by the total energy intake. A meta-analysis suggested that children with obesity to have significantly different markers of Fe deficiency than those in control group [21]. Specially, children with obesity had significantly lower Fe, transferrin saturation, and total-iron binding capacity along with higher ferritin, soluble transferrin receptors and hepcidin-25 than children of normal weight [21]. In this study, we similarly discovered higher level of dietary Fe intake was associated with lower odds of childhood obesity, but the underlying mechanism is needed further exploration. A cross-sectional study based on the NHANES database by Zhao et al. [22] showed that the highest quartile of Na intake was positively associated with overweight, obesity, and central obesity among children in the United States. In the same way, results of the current study found the relationships between higher dietary intake level of Na and childhood obesity as well as higher BMI in children. Besides, Wang et al. [23] discovered whole-blood Mg concentration was an independent risk factor of overweight or obesity in youngsters. Cai et al. [24] indicated that high dietary K intake could not reduce the risk of obesity, while serum K and urinary Na-to-K ratio was associated with obesity. In our study, the average dietary consumptions of Ca, Fe, Zn and Cu were significantly lower in children with obesity than those without obesity, and consumptions of Na and Se were higher in children with obesity. The possible reason for these inconsistent results, we speculated, may be that children with obesity are more likely to control the quantity of dietary minerals intakes, such as Cu and Ca, following medical advices. Moreover, the specific mechanisms on associations between dietary intakes of minerals are needed to be further clarified.

Obesity is characterized by a low-grade systemic chronic inflammatory state [25]. Constant macrophage infiltrate into adipose tissue and the local pro-inflammatory cytokines change in obesity may lead to impaired erythropoietin production and altered response of erythroid precursors, which has been a recognized mechanism of anemia associated with chronic diseases [4,26]. Studies have reported that lower serum Fe concentrations and transferrin saturation were found in obese/overweight individuals, and a negative correlation between transferrin saturation and adiposity was also found in school children with obesity who aged between 9 and 13 years old [27,28]. In the current study, we are unavailable to obtain the anemia-related indexes such as serum Fe and transferrin saturation between children with obesity and without obesity. Basing on a previous study, we speculated that lack of dietary Fe may disturb the hemoproteins or other non-heme proteins metabolism, and affect the formation of toxic oxygen free radicals that further influence the development of obesity [29]. Analogously, Zn is an efficient antioxidant and plays a major role in some protein productions such as insulin action and insulin receptor tyrosine kinase activity [30]. We found that higher dietary Zn intake was associated with obesity and lower BMI in children. The underlying biological mechanism could be the gene expression of Zn-a2-glycoprotein (ZAG) is lower in subcutaneous and visceral adipose tissue and livers of obese individuals, which may play an important role in the development of obesity [31]. Up to now, the potential mechanisms on a direct association between dietary Na intake and obesity are still unclear. Epidemiologic studies suggested that salt intake was associated with leptin concentrations, percentage of body fat, and adipose tissue [32,33]. High-salt diets may contribute to the progression of obesity by increasing fasting ghrelin, which regulates appetite, glucose homeostasis, and fat deposition [34]. Whether these mechanisms are appropriate for the explanation on our findings are needed further basic research to verify. In addition, Cu is a redox-active metal, and elevated Cu in circulation may contribute to exacerbate oxidative stress by generating free radical species, thereby inducing lipid peroxidation. In this context, we suggested that the abnormal lipid profiles associated with Cu overload may lead to the obesity related systemic inflammation and oxidative stress [16,35].

The associations between higher dietary intake of Zn and lower odds of childhood obesity, as well as between higher dietary intakes of Mg and Na and higher odds of childhood obesity were also found in children aged 6–11 years old. The negative relationship between dietary Fe intake and BMI was found in children who aged 2–5 years old, and 12–17 years old. The negative relationship between dietary Zn intake and BMI was only found in those who aged 6–11 years old. Also, positive association between dietary Na intake and BMI was found in children who aged from 6 to 17 years old. For children with different age, it is necessary to develop individualization recommendations of minerals intake in obesity. After 2 years old, not only dietary patterns (including minerals consumption), but also sugar-sweetened beverages consumption, eating behavior, meal frequency and composition, portion size and physical activity, and sedentary behavior were the influencing factors of childhood obesity [6,36]. To prevent and manage children with overweight/obesity is a long-term, multi-stage process. A balanced diet with fiber-rich foods including whole grains, lentils, nuts, fruits and vegetables benefits to improve and prevent obesity [37]. Combining our findings, children aged 2–5 years old are recommended to get plenty of iron-containing fruits and vegetables, whereas children aged 6–11 years old are recommended for foods are rich in Zn. Snacks with high Na content should be strictly controlled at all ages during childhood, and a regular meal pattern along with healthy habits were also needed [38]. In addition to dietary, children should optimally have 60 mins of moderate to vigorous physical activity, at least 5 days per week, to decrease the risk of developing obesity [39]. Besides, school-age children and adolescents should sleep for 8 to 11 hours per day and in quiet surroundings, due to insufficient sleep can affect dietary intake and metabolism, which may lead to obesity [40].

This study population was from the NHANES database, so that the sample size was large and were the representative population in the United States. We explored the association between dietary intakes of nine common minerals and childhood obesity and BMI, which may provide some references for further studies exploring the causal associations, and may further help the prevention and management of childhood obesity. However, there are some limitations in this study. This is a retrospective study so that no causal association could be concluded. Information of dietary intake were collected using the 24-hour recall, which can cause the recalling bias, and at the same time, could not reflect the long-term dietary habits. Therefore, further prospective cohort studies focusing on the long-term effects of dietary minerals intake on childhood obesity are still needed.

## Conclusion

Dietary Fe and Zn intakes may benefit to reduce the odds of childhood obesity, whereas the dietary Cu and Na consumptions should be controlled suitably. In addition, it is necessary to develop individualization recommendations of minerals intake for children with high-risk of obesity in different age in the future.

## Supporting information

S1 Checklist STROBE statement—Checklist of items that should be included in reports of observational studies. (DOCX)

## References

[1] Verduci E, Di Profio E, Fiore G, Zuccotti G. Integrated Approaches to Combatting Childhood Obesity. Ann Nutr Metab. 2022;78 Suppl 2:8–19. Epub 2022/06/10. doi: 10.1159/000524962 . 35679843

[2] Gomahr J, Julian V, Thivel D, Maruszczak K, Schneider AM, Weghuber D. Childhood obesity prevention: what can be achieved? Curr Opin Clin Nutr Metab Care. 2022;25(3):223–31. Epub 2022/03/09. doi: 10.1097/MCO.0000000000000831 . 35256565

[3] Apperley LJ, Blackburn J, Erlandson-Parry K, Gait L, Laing P, Senniappan S. Childhood obesity: A review of current and future management options. Clin Endocrinol (Oxf). 2022;96(3):288–301. Epub 2021/11/10. doi: 10.1111/cen.14625 . 34750858

[4] Gonzalez-Dominguez A, Visiedo-Garcia FM, Dominguez-Riscart J, Gonzalez-Dominguez R, Mateos RM, Lechuga-Sancho AM. Iron Metabolism in Obesity and Metabolic Syndrome. Int J Mol Sci. 2020;21(15). Epub 2020/08/06. doi: 10.3390/ijms21155529 . 32752277 PMC7432525

[5] The Lancet Diabetes E. Childhood obesity: a growing pandemic. Lancet Diabetes Endocrinol. 2022;10(1):1. Epub 2021/12/06. doi: 10.1016/S2213-8587(21)00314-4 . 34863372 PMC9765420

[6] Verduci E, Bronsky J, Embleton N, Gerasimidis K, Indrio F, Koglmeier J, et al. Role of Dietary Factors, Food Habits, and Lifestyle in Childhood Obesity Development: A Position Paper From the European Society for Paediatric Gastroenterology, Hepatology and Nutrition Committee on Nutrition. J Pediatr Gastroenterol Nutr. 2021;72(5):769–83. Epub 2021/03/16. doi: 10.1097/MPG.0000000000003075 . 33720094 PMC9770153

[7] Bucher Della Torre S, Keller A, Laure Depeyre J, Kruseman M. Sugar-Sweetened Beverages and Obesity Risk in Children and Adolescents: A Systematic Analysis on How Methodological Quality May Influence Conclusions. J Acad Nutr Diet. 2016;116(4):638–59. Epub 2015/07/22. doi: 10.1016/j.jand.2015.05.020 . 26194333

[8] Fabiano V, Albani E, Cammi GM, Zuccotti GV. Nutrition in developmental age: few rules to stay healthy. Minerva Pediatr. 2020;72(3):182–95. Epub 2020/04/11. doi: 10.23736/S0026-4946.20.05803-X . 32274912

[9] Amin MN, Siddiqui SA, Uddin MG, Ibrahim M, Uddin SMN, Adnan MT, et al. Increased Oxidative Stress, Altered Trace Elements, and Macro-Minerals Are Associated with Female Obesity. Biol Trace Elem Res. 2020;197(2):384–93. Epub 2020/01/07. doi: 10.1007/s12011-019-02002-z . 31902098

[10] Barra NG, Anhe FF, Cavallari JF, Singh AM, Chan DY, Schertzer JD. Micronutrients impact the gut microbiota and blood glucose. J Endocrinol. 2021;250(2):R1–R21. Epub 2021/06/25. doi: 10.1530/JOE-21-0081 . 34165440

[11] Smita RM, Shuvo APR, Raihan S, Jahan R, Simin FA, Rahman A, et al. The Role of Mineral Deficiencies in Insulin Resistance and Obesity. Curr Diabetes Rev. 2022;18(7):e171121197987. Epub 2021/11/19. doi: 10.2174/1573399818666211117104626 . 34789132

[12] Dubey P, Thakur V, Chattopadhyay M. Role of Minerals and Trace Elements in Diabetes and Insulin Resistance. Nutrients. 2020;12(6). Epub 2020/06/27. doi: 10.3390/nu12061864 . 32585827 PMC7353202

[13] Zhang X, Wang J, Li J, Yu Y, Song Y. A positive association between dietary sodium intake and obesity and central obesity: results from the National Health and Nutrition Examination Survey 1999–2006. Nutr Res. 2018;55:33–44. Epub 2018/06/20. doi: 10.1016/j.nutres.2018.04.008 . 29914626

[14] Lanaspa MA, Kuwabara M, Andres-Hernando A, Li N, Cicerchi C, Jensen T, et al. High salt intake causes leptin resistance and obesity in mice by stimulating endogenous fructose production and metabolism. Proc Natl Acad Sci U S A. 2018;115(12):3138–43. Epub 2018/03/07. doi: 10.1073/pnas.1713837115 . 29507217 PMC5866545

[15] Gu K, Xiang W, Zhang Y, Sun K, Jiang X. The association between serum zinc level and overweight/obesity: a meta-analysis. Eur J Nutr. 2019;58(8):2971–82. Epub 2018/12/14. doi: 10.1007/s00394-018-1876-x . 30542939

[16] Gu K, Li X, Xiang W, Jiang X. The Relationship Between Serum Copper and Overweight/Obesity: a Meta-analysis. Biol Trace Elem Res. 2020;194(2):336–47. Epub 2019/07/14. doi: 10.1007/s12011-019-01803-6 . 31300957

[17] Fulgoni K, Fulgoni VL 3rd. Trends in Total, Added, and Natural Phosphorus Intake in Adult Americans, NHANES 1988–1994 to NHANES 2015–2016. Nutrients. 2021;13(7). Epub 2021/07/03. doi: 10.3390/nu13072249 . 34210102 PMC8308364

[18] Blanton CA, Moshfegh AJ, Baer DJ, Kretsch MJ. The USDA Automated Multiple-Pass Method accurately estimates group total energy and nutrient intake. J Nutr. 2006;136(10):2594–9. Epub 2006/09/22. doi: 10.1093/jn/136.10.2594 . 16988132

[19] Li R, Xia J, Zhang XI, Gathirua-Mwangi WG, Guo J, Li Y, et al. Associations of Muscle Mass and Strength with All-Cause Mortality among US Older Adults. Med Sci Sports Exerc. 2018;50(3):458–67. Epub 2017/10/11. doi: 10.1249/MSS.0000000000001448 . 28991040 PMC5820209

[20] Fan Y, Zhang C, Bu J. Relationship between Selected Serum Metallic Elements and Obesity in Children and Adolescent in the U.S. Nutrients. 2017;9(2). Epub 2017/02/07. doi: 10.3390/nu9020104 . 28165362 PMC5331535

[21] Malden S, Gillespie J, Hughes A, Gibson AM, Farooq A, Martin A, et al. Obesity in young children and its relationship with diagnosis of asthma, vitamin D deficiency, iron deficiency, specific allergies and flat-footedness: A systematic review and meta-analysis. Obes Rev. 2021;22(3):e13129. Epub 2020/08/19. doi: 10.1111/obr.13129 . 32808447 PMC7611974

[22] Zhao L, Ogden CL, Yang Q, Jackson SL, Loria CM, Galuska DA, et al. Association of Usual Sodium Intake with Obesity Among US Children and Adolescents, NHANES 2009–2016. Obesity (Silver Spring). 2021;29(3):587–94. Epub 2021/02/03. doi: 10.1002/oby.23102 . 33528899 PMC9134125

[23] Wang T, Wang L, Ma N, Gu S, Jiang D, Li J, et al. Whole-blood magnesium and blood lipids are individually and jointly associated with an elevated likelihood of youngsters being overweight or obese: A matched case-control study using the propensity score. Nutrition. 2022;93:111425. Epub 2021/09/05. doi: 10.1016/j.nut.2021.111425 . 34481288

[24] Cai X, Li X, Fan W, Yu W, Wang S, Li Z, et al. Potassium and Obesity/Metabolic Syndrome: A Systematic Review and Meta-Analysis of the Epidemiological Evidence. Nutrients. 2016;8(4):183. Epub 2016/03/31. doi: 10.3390/nu8040183 . 27023597 PMC4848652

[25] Schmidt FM, Weschenfelder J, Sander C, Minkwitz J, Thormann J, Chittka T, et al. Inflammatory cytokines in general and central obesity and modulating effects of physical activity. PLoS One. 2015;10(3):e0121971. Epub 2015/03/18. doi: 10.1371/journal.pone.0121971 . 25781614 PMC4363366

[26] Ferrucci L, Guralnik JM, Woodman RC, Bandinelli S, Lauretani F, Corsi AM, et al. Proinflammatory state and circulating erythropoietin in persons with and without anemia. Am J Med. 2005;118(11):1288. Epub 2005/11/08. doi: 10.1016/j.amjmed.2005.06.039 . 16271918

[27] Zhao L, Zhang X, Shen Y, Fang X, Wang Y, Wang F. Obesity and iron deficiency: a quantitative meta-analysis. Obes Rev. 2015;16(12):1081–93. Epub 2015/09/24. doi: 10.1111/obr.12323 . 26395622

[28] Manios Y, Moschonis G, Chrousos GP, Lionis C, Mougios V, Kantilafti M, et al. The double burden of obesity and iron deficiency on children and adolescents in Greece: the Healthy Growth Study. J Hum Nutr Diet. 2013;26(5):470–8. Epub 2013/01/03. doi: 10.1111/jhn.12025 . 23279448

[29] Yiannikourides A, Latunde-Dada GO. A Short Review of Iron Metabolism and Pathophysiology of Iron Disorders. Medicines (Basel). 2019;6(3). Epub 2019/08/08. doi: 10.3390/medicines6030085 . 31387234 PMC6789448

[30] Beletate V, El Dib RP, Atallah AN. Zinc supplementation for the prevention of type 2 diabetes mellitus. Cochrane Database Syst Rev. 2007;(1):CD005525. Epub 2007/01/27. doi: 10.1002/14651858.CD005525.pub2 . 17253560

[31] Selva DM, Lecube A, Hernandez C, Baena JA, Fort JM, Simo R. Lower zinc-alpha2-glycoprotein production by adipose tissue and liver in obese patients unrelated to insulin resistance. J Clin Endocrinol Metab. 2009;94(11):4499–507. Epub 2009/07/23. doi: 10.1210/jc.2009-0758 . 19622624

[32] Libuda L, Kersting M, Alexy U. Consumption of dietary salt measured by urinary sodium excretion and its association with body weight status in healthy children and adolescents. Public Health Nutr. 2012;15(3):433–41. Epub 2011/09/21. doi: 10.1017/S1368980011002138 . 21929845

[33] Larsen SC, Angquist L, Sorensen TI, Heitmann BL. 24h urinary sodium excretion and subsequent change in weight, waist circumference and body composition. PLoS One. 2013;8(7):e69689. Epub 2013/08/13. doi: 10.1371/journal.pone.0069689 . 23936079 PMC3723894

[34] Zhang Y, Li F, Liu FQ, Chu C, Wang Y, Wang D, et al. Elevation of Fasting Ghrelin in Healthy Human Subjects Consuming a High-Salt Diet: A Novel Mechanism of Obesity? Nutrients. 2016;8(6). Epub 2016/05/31. doi: 10.3390/nu8060323 . 27240398 PMC4924164

[35] Ma J, Xie Y, Zhou Y, Wang D, Cao L, Zhou M, et al. Urinary copper, systemic inflammation, and blood lipid profiles: Wuhan-Zhuhai cohort study. Environ Pollut. 2020;267:115647. Epub 2020/12/02. doi: 10.1016/j.envpol.2020.115647 . 33254652

[36] Sarni ROS, Kochi C, Suano-Souza FI. Childhood obesity: an ecological perspective. J Pediatr (Rio J). 2022;98 Suppl 1(Suppl 1):S38–S46. Epub 2021/11/16. doi: 10.1016/j.jped.2021.10.002 . 34780713 PMC9510906

[37] Cathaoir KO. Childhood Obesity and the Right to Health. Health Hum Rights. 2016;18(1):249–62. Epub 2016/10/27. . 27781014 PMC5070695

[38] Cuda SE, Censani M. Pediatric Obesity Algorithm: A Practical Approach to Obesity Diagnosis and Management. Front Pediatr. 2018;6:431. Epub 2019/02/08. doi: 10.3389/fped.2018.00431 . 30729102 PMC6351475

[39] Styne DM, Arslanian SA, Connor EL, Farooqi IS, Murad MH, Silverstein JH, et al. Pediatric Obesity-Assessment, Treatment, and Prevention: An Endocrine Society Clinical Practice Guideline. J Clin Endocrinol Metab. 2017;102(3):709–57. Epub 2017/03/31. doi: 10.1210/jc.2016-2573 . 28359099 PMC6283429

[40] Golley RK, Maher CA, Matricciani L, Olds TS. Sleep duration or bedtime? Exploring the association between sleep timing behaviour, diet and BMI in children and adolescents. Int J Obes (Lond). 2013;37(4):546–51. Epub 2013/01/09. doi: 10.1038/ijo.2012.212 . 23295498

