# Supplementary material for: Association between minerals intake and childhood obesity: A cross-sectional study of the NHANES database in 2007–2014
Source: PLoS One. 2023 Dec 27;18(12):e0295765. doi: 10.1371/journal.pone.0295765 (PMC10752540; doi:10.1371/journal.pone.0295765)
Supplement: Attachment — Submitted filename: Response to Reviewers.docx [file pone.0295765.s003.docx]

**Responses to Reviewers**

Dear Editor and reviewers,

We highly appreciate and thank the Editors and the reviewers for your time and valuable comments on this manuscript. We have carefully studied the comments and suggestions and revised our paper accordingly. The following are our point-by-point responses to the comments. We hope that the revisions are acceptable and that our responses adequately address the comments. Thank you for your consideration.

**Responses to Reviewer #1’s comments**

This manuscript is still lacking in areas related to coherency and grammar. From the Abstract to the Conclusion there are still areas that need to be written better. It appears that the listed changes were made but that the overall manuscript was not fully edited. I also feel that the discussion section is not as approachable as it needs to be. Readers need to be able to fully understand both the results and the contribution after reading the discussion.

Response: Thank you for your valuable comments. We have reviewed and revised the manuscript to make it more coherent and easier to understand. We also invited a native English-speaker to help us revise, and we hope the revised manuscript could achieve your standards.

**Responses to Reviewer #2’s comments**

1 In section of “Statistical analysis”: you should mention clearly which reference group in the dependent variable is

Response: We have added the description on reference group.

2 Table 4 and table 5:

Round numbers to 2 decimal places throughout the manuscript to keep consistent.

Response: Thank you for your valuable comment. We have revised the tables to keep the numbers to 2 decimal places consistently.

3 The authors should improve the scientific writing and check text carefully before submission:

line 120 formula: weight/Height2 (kg/m2): should be “height”

line 124 that ≥95th percentile indicates obesity: should be “that of…”

line 174 obesity? are showed in Table 1: display incorrectly

line 176 If you decided to use comma for numbers with 4 or more digits, please be consistent throughout the manuscript

Response: Thank you for your valuable comments. The above mistakes have been revised, and we have checked and edited the manuscript.
